# Mozart K.448 listening decreased seizure recurrence and epileptiform discharges in children with first unprovoked seizures: a randomized controlled study

**DOI:** 10.1186/1472-6882-14-17

**Published:** 2014-01-13

**Authors:** Lung-Chang Lin, Mei-Wen Lee, Ruey-Chang Wei, Hin-Kiu Mok, Rei-Cheng Yang

**Affiliations:** 1 Department of Pediatrics, School of Medicine, College of Medicine, Kaohsiung Medical University, Kaohsiung City 80708, Taiwan; 2 Department of Pediatrics, Kaohsiung Medical University Hospital, Kaohsiung Medical University, #100, Tzu-you 1st Road, Kaohsiung City 80708, Taiwan; 3 Department of Music, National Sun Yat-Sen University, Kaohsiung City 804, Taiwan; 4 Institute of Applied Physics and Underseas Technology, National Sun Yat-Sen University, Kaohsiung City 804, Taiwan; 5 Institute of Marine Biology, National Sun Yat-Sen University, Kaohsiung City 804, Taiwan; 6 Department of Pediatrics, Changhua Christian Hospital, Changhua, Taiwan

**Keywords:** Mozart K.448, First unprovoked seizure, Epileptiform discharges, Music therapy, Children

## Abstract

**Background:**

Increasing numbers of reports show the beneficial effects of listening to Mozart music in decreasing epileptiform discharges as well as seizure frequency in epileptic children. There has been no effective method to reduce seizure recurrence after the first unprovoked seizure until now. In this study, we investigated the effect of listening to Mozart K.448 in reducing the seizure recurrence rate in children with first unprovoked seizures.

**Methods:**

Forty-eight children who experienced their first unprovoked seizure with epileptiform discharges were included in the study. They were randomly placed into treatment (n = 24) and control (n = 24) groups. Children in the treatment group listened to Mozart K.448 daily before bedtime for at least six months. Two patients in the treatment group were excluded from analysis due to discontinuation intervention. Finally, forty-six patients were analyzed. Most of these patients (89.1%) were idiopathic in etiology. Seizure recurrence rates and reduction of epileptiform discharges were compared.

**Results:**

The average follow-up durations in the treatment and control groups were 18.6 ± 6.6 and 20.1 ± 5.1 months, respectively. The seizure recurrence rate was estimated to be significantly lower in the treatment group than the control group over 24 months (37.2% vs. 76.8%, *p* = 0.0109). Significant decreases in epileptiform discharges were also observed after 1, 2, and 6 months of listening to Mozart K.448 when compared with EEGs before listening to music. There were no significant differences in gender, mentality, seizure type, and etiology between the recurrence and non-recurrence groups.

**Conclusions:**

Although the case number was limited and control music was not performed in this study, the study revealed that listening to Mozart K.448 reduced the seizure recurrence rate and epileptiform discharges in children with first unprovoked seizures, especially of idiopathic etiology. We believe that Mozart K.448 could be a promising alternative treatment in patients with first unprovoked seizures and abnormal EEGs. Further large-scaled study should be conducted to confirm the effect.

**Trial registration:**

NCT01892605, date: June-19-2013

## Background

Music has been used for healing mental and physical diseases. Rauscher et al. report that brief exposure to Mozart’s Sonata for Two Pianos in D major, K.448 (Mozart K.448), produces a temporal increase in spatial reasoning scores [1], the so-called “Mozart Effect.” In addition to improvement of cognitive function, subsequent studies reveal positive effects from listening to music for many medical diseases, including hypertension, anxiety, and dementia [2]–[4]. For example, Särkämö et al. investigated patients with strokes who listened to their favorite music for two months. Their results show that recovery in the domains of verbal memory and focus attention improved significantly more in the music group than in the control group, even six months after the stroke [5]. In patients with Parkinson disease, after listening to self-selected music, motor coordination with “Vienna Test System” shows improvement in aiming and line tracking [6]. This study provides evidence that specific music can improve the precision of arm and finger movement. Relaxing classical music was also used in sleep disorders. 94 students with sleep complaints participated a music study. They listened to relaxing classical music, including some popular pieces from Baroque to Romantic, for 45 minutes every night at bedtime for 3 consecutive weeks, or audiobooks-a CD containing 11 hours of short stories by Hungarian writers such as Frigyes Karinthy, Gyula Krúdy, Géza Gárdonyi, Zsigmond Móricz and Mihály Babits for 45 minutes every night at bedtime for 3 consecutive weeks, or no intervention for 3 weeks. The results show music significantly improves sleep quality according to the Pittsburg sleep quality index and depressive symptoms. However, sleep quality and depressive symptoms did not improve in the audiobook and control group [7]. Regarding epilepsy, Hughes et al. and our previous study show that epileptiform discharges decrease when listening to Mozart K.448 in patients with epilepsy [8,9]. Furthermore, our study also reveals that listening to Mozart K.448 reduces seizure frequencies in children with intractable seizures [10].

The seizure recurrence rate after the first unprovoked seizure in pediatric patients is highly variable, depending on the follow-up period and patient selection. Age at onset of epilepsy under 1 year, remote symptomatic etiology, developmental delay/mental retardation, abnormal EEG background, frequent epileptiform discharges, and abnormal neuroimaging are all significant predictors of a higher risk for recurrence of epilepsy [11,12]. Children with a recurrence have a similar epileptic outcome when compared to children presenting with multiple seizures, regardless whether they were treated after the first unprovoked seizure or not [13]. This argues in favor of withholding anti-epileptic drug treatment at least until a second seizure has occurred to reduce the potential for adverse effects of drugs, including unpleasant physical effects, adverse cognitive and behavioral changes, and potential teratogenicity [13,14]. Until now, there has been no effective method to reduce seizure recurrence after the first unprovoked seizure. In this study, we investigated the effect of Mozart K.448 on the seizure recurrence in children with their first unprovoked seizure who have epileptiform discharges.

## Methods

### Subjects

#### Inclusion criteria and exclusion criteria

Children less than 18 years of age with first unprovoked seizure were investigated in the out-patient department of Kaohsiung Medical University Hospital. Patients with epileptiform EEGs were included in this study. Patients who (1) had neurodegenerative diseases, or (2) did not have epileptiform discharges were excluded. Participants in the study received EEG examinations after their first seizure occurred. Each EEG was recorded digitally (Harmonie DVN V5.1, Montreal, Canada). Electrodes were placed according to the International 10–20 System and two neurologists reviewed the EEG recordings. Informed consent was given by a family member or legal guardian in each case. This study was approved by the Institutional Review Board of Kaohsiung Medical University Hospital (KMUH-IRB-970391).

#### Music listening and evaluating seizure recurrence

This randomized controlled study was conducted in Kaohsiung Medical University Hospital. The patients in this study were randomly placed in the control or treatment group by the chief investigator in a 1:1 ratio, by randomly generating even (for treatment) and uneven (for control) numbers using a computer program (http://www.random.org). No stratification for age, sex, or seizure type was performed. The patients in the study did not receive AED after their first unprovoked seizure. The treatment group listened to the first movement of Mozart K.448 for eight minutes once daily before bedtime for at least six months. None of the participants had reported listening to Mozart K.448 music in the past. The control group did not receive any music. All of the patients received follow-up telephone calls monthly. Patients who experienced seizure recurrence were advised to begin anti-epileptic drug treatment. All the data were collected at the Kaohsiung Medical University Hospital from April 2009 to December 2011.

#### Follow up EEG

Patients who listened to Mozart K.448 without seizure recurrence received EEG examinations before, and after 1, 2, and 6 months of listening. Patients in the control group received EEG examinations at the beginning of study and before anti-epileptic drug treatment. The epileptiform discharges were defined as distinct waves or complexes, distinguished from background activity. Two pediatric neurologists analyzed these EEGs and calculated the frequency of epileptiform discharges by blind. The total number of epileptiform discharges during each section (before, and after 1, 2, and 6 months of listening) were divided by the duration (in minutes) of the section and compared. Percentage changes in epileptiform discharge were calculated as ((the discharge before music listening - discharge after music listening)/discharge before music listening) × 100. All recordings were performed during the daytime. To decrease the factors influencing epileptiform discharges, each patient maintained the same state of wakefulness during each recording period.

#### Statistical analysis

Seizure recurrence rates were determined by Kaplan-Meier estimates, defining an event as the recurrence of a second seizure. Sample size for Kaplan-Meier approach was estimated by the survival power calculator (http://www.statstodo.com/SSizSurvival_Pgm.php). The follow-up time for patients with seizure recurrence was from the beginning of music treatment to the last clinical visit. Differences in the distribution of treatment and control groups were calculated using the Chi-square test. The paired t-test and ANOVA were used to compare the percentage of epileptiform discharge reduction at the 1^st^, 2^nd^, and 6^th^ month of music listening. A p value less than 0.05 was considered significant.

## Results

**Figure 1:**
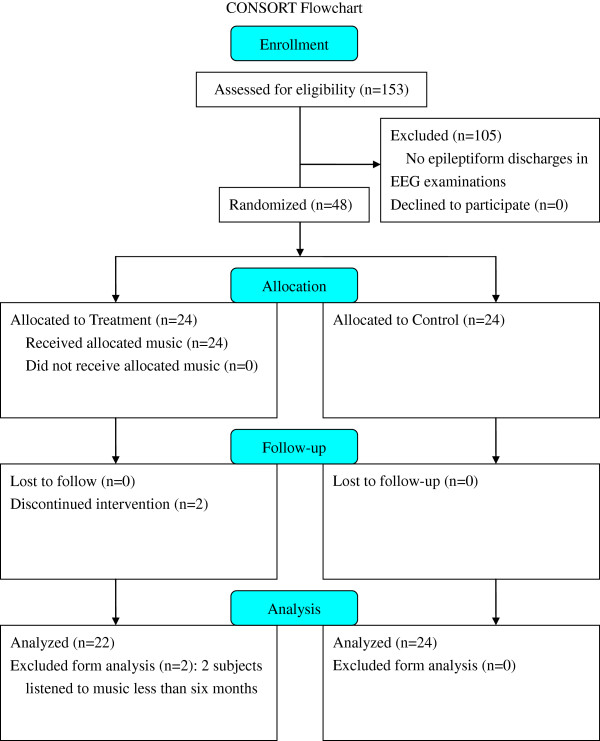
The patient recruitment flowchart.

153 patients with their first unprovoked seizure were investigated for inclusion in this study and 107 patients were excluded from analysis. No epileptiform discharges were found in 105 patients. Forty-eight patients with epileptiform discharges were enrolled. Twenty-four patients were placed in the treatment group and 24 patients in the control group (Figure 1). Two patients who listened to music less than six months in the treatment group were excluded. Finally, forty-six patients were analyzed. The mean age of the treatment group was 9 years 6 months ± 3 years 10 months, and the control group was 8 years 7 months ± 3 years 10 months. Forty-three patients demonstrated normal intelligence, two patients had a reduced IQ, and one patient had an undetermined IQ level. The majority of patients (n = 41) were idiopathic in etiology, and five patients were symptomatic (Table 1). There were no significant differences in gender, mentality, seizure type, and etiology between the treatment and control groups. None of the patients suffered from musicogenic epilepsy at the time of study.

**Table 1:** Profile comparison between treatment with Mozart K. 448 and control group

	**Treatment n (%)**	**Control n (%)**	**p-value**
**Sex**			
Male	13 (59.1)	12 (50)	0.536
Female	9 (40.9)	12 (50)	
**Age**	9 y 6 m ± 3 y 10 m	8 y 7 m ± 3 y 10 m	0.413
**Mentality**			
IQ≥70	20 (90.9)	23 (95.8)	0.209
IQ<70	2 (9.1)	0 (0)	
undetermined	0 (0)	1 (4.2)	
**Seizure type**			
Generalized	4 (18.2)	7 (29.2)	0.383
Focal	18 (81.2)	17 (65)	
**Etiology**			
Idiopathic	19 (83.4)	22 (89.1)	0.564
Symptomatic	3 (16.6)	2 (10.9)	

### Seizure recurrence after music listening

**Figure 2:**
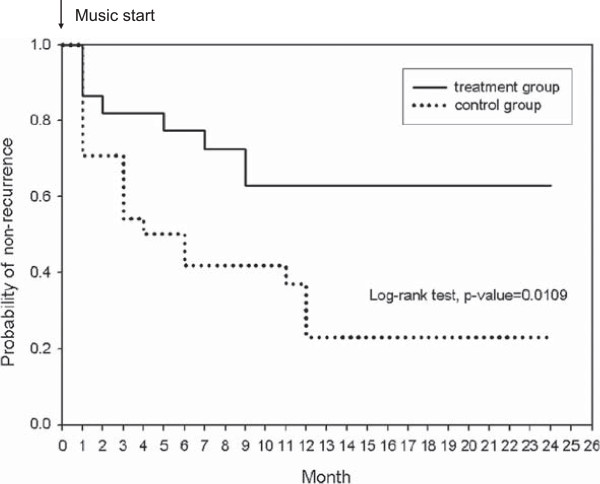
**The seizure recurrence rates after the first unprovoked seizure between treatment and control groups.** The seizure recurrence rate was estimated to be significantly lower in the treatment group than the control group by 24 months (*p* = 0.0109).

The average follow-up durations in the treatment group and control group were 18.6 ± 6.6 (range from 6 to 24 months) and 20.1 ± 5.1 (range from 10 to 24 months) months, respectively. During the follow-up period, 8 of 22 patients in the treatment group had seizure recurrence, while 18 of 24 patients in the control group had seizure recurrence. After at least six months of Mozart K.448 listening, the seizure recurrence rate in the treatment group at the 6 months follow-up was 22.7% and was estimated to be 37.2% by 12 and 24 months. In the control group, at the 6 months follow-up, the seizure recurrence rate was 58.3% and was estimated to be 76.8% by 12 and 24 months. A significantly higher recurrence rate was noted in the control group than the treatment group (p = 0.0109) (Figure 2). There were no significant differences in gender, mentality, seizure type, and etiology between the recurrence and non-recurrence subjects in either treatment or control group (Table 2).

**Table 2:** **Profile comparison between recurrence and non**-**recurrence group**

	**Treatment**			**Control**		
	**Recurrence**	**Non-recurrence**	**p**-**value**	**Recurrence**	**Non-recurrence**	**p**-**value**
	**n (%)**	**n (%)**		**n (%)**	**n (%)**	
**Sex**						
Male	3 (37.5)	10 (71.4)	0.119	10 (55.6)	2 (33.3)	0.346
Female	5 (62.5)	4 (28.6)		8 (44.4)	4 (66.7)	
**Mentality**						
IQ≥70	8 (100)	12 (85.7)	0.262	17 (94.4)	6 (100)	0.555
IQ<70	0 (0)	2 (14.3)		0 (0)	0 (0)	
undetermined	0 (0)	0 (0)		1 (5.6)	0 (0)	
**Seizure type**						
Generalized	2 (25)	2 (14.3)	0.531	4 (22.2)	3 (50)	0.195
Focal	6 (75)	12 (85.7)		14 (77.8)	3 (50)	
**Etiology**						
Idiopathic	7 (87.5)	12 (85.7)	0.906	16 (88.9)	6 (100)	0.394
Symptomatic	1 (12.5)	2 (14.3)		2 (11.1)	0 (0)	

### Changes in frequency of epileptiform discharges in the treatment and control groups

**Figure 3:**
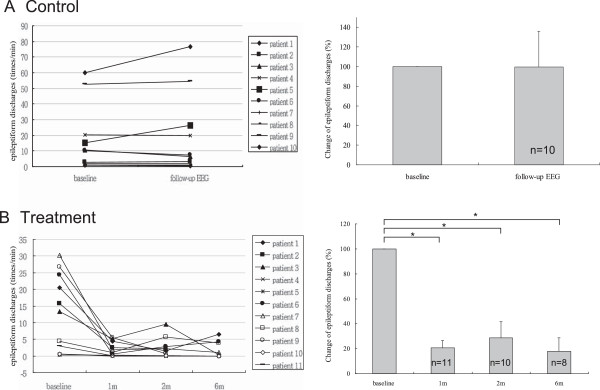
**The comparison of reduction of epileptiform discharges in absolute frequency and percentage of reduction between treatment and control groups.** There was no significant difference between baseline and follow-up (the interval between baseline and follow-up EEGs ranged from two to seven months) epileptiform discharge frequencies in the control group **(A)**. Significant decreases in epileptiform discharges were observed after 1, 2, and 6 months of listening to Mozart K.448 when compared with EEGs before listening to music **(B)**. * *p* < 0.05.

There was no significant difference between baseline and follow-up (the interval between baseline and follow-up EEGs ranged from two to seven months) epileptiform discharge frequencies in the control group (3A). Fourteen patients did not experience seizure recurrence during any of the follow-up periods in the treatment group. Among them, eleven patients received EEG follow-ups after1 month, ten patients after 2 months, and eight patients after 6 months of listening to Mozart K.448. The absolute frequency of epileptiform discharges demonstrated a trend of decrease after music exposure (Figure 3B). Since the average epileptiform discharge frequency in each patient of treatment group before Mozart K.448 listening was highly variable, ranging from 0.3/min to 30.2/min, the change in epileptiform discharges was expressed by percentage of reduction. Significant decreases in epileptiform discharges were found after 1, 2, and 6 months of listening to Mozart K.448 when compared with EEGs before listening to music (decreased by 79.4 ± 20.0%, 71.2 ± 40.3%, and 82.1 ± 30.6% respectively, *p* < 0.001). However, the decreases of epileptiform discharges did not show a significant difference between 1, 2, and 6 months of listening to Mozart K.448 (Figure 3B). On the contrary, during the average period of 3.3 months (ranged from 2 to 7 months), ten patients in the control group received EEG follow-ups, and there was no significant change in epileptiform discharges between EEGs at the beginning of study and before anti-epileptic drug treatment (increased by 0.3 ± 36.4%, *p* =0.310) (Figure 3A).

## Discussion

Mozart K.448 listening is reported to diminish the intensity of tinnitus, improve the paper-folding and cutting tests in patients with mild cognitive impairment, and increase weight gain in preterm infants by reducing resting energy expenditure [15]–[17]. Regarding epilepsy, our previous works show that interictal discharges were reduced in most patients with epilepsy when they listened to Mozart K.448 [9]. In addition, 72.7% of the patients with refractory epilepsy became seizure free or had a very good response by listening to Mozart K.448 [10]. Our previous report also demonstrates that Mozart K.448 is not the only piece of music to have beneficial effects on children with epilepsy, and that listening to Mozart K.545 with similar lower harmonics can decrease epileptiform discharges in epileptic children as well [18].

In this current study, we investigated the effect of Mozart K.448 on seizure recurrence after the first unprovoked seizure. The results showed that listening to Mozart K.448 once daily reduced the seizure recurrence rate and epileptiform discharges. The estimated seizure recurrence rate in the control group was 76.8%, while it was 37.2% in music treated patients. The epileptiform discharges also showed an approximate 70-80% reductions after 1, 2, and 6 months of music listening.

Seizure recurrence after the first unprovoked seizure in pediatric patients ranges from 26-71% [19]. Risk factors for seizure recurrence include a remote symptomatic etiology, an abnormal EEG, a seizure occurring while asleep, a history of prior febrile seizures, and Todd’s paresis [12]. In a large study, 564 patients, including adults and children who had first unprovoked seizures, have been followed up for 2–4 years. Sixty-seven percent of them had a recurrence within 12 months of the first seizure, and 78% had a recurrence within 36 months [20]. In the under the age of 16 group, the seizure recurrence rate was 83% by 36 months [20]. In our study, the seizure recurrence rate was estimated to be 76.8% by 24 months in the control group. An abnormal EEG and an age less than 16 appeared to be risk factors for a higher recurrence rate in our study. However, the seizure recurrence rate did not demonstrate a significant difference in patients with different gender, mentality, seizure type, and etiology. The results were similar to our previous studies which show that gender, mentality, and etiology of epilepsy do not influence the short-term or long-term music effect on epileptiform discharges [9,21].

The epileptiform discharges were significantly reduced in EEGs performed 1, 2, and 6 months after initiating music listening in patients without seizure recurrence. However, the epileptiform discharges did not decrease in a duration-dependence manner. EEG improvement after one month of listening to Mozart K.448 may serve as an indicator in determining the long term outcome of music intervention. Although it is not possible to predict how long the patients should be treated, the effectiveness of music listening was demonstrable within one month and continued for at least 6 months. We suggest that listening to music daily for 6 months has a beneficial effect in decreasing seizure recurrence in children with first unprovoked seizures.

Recently, several theories have been introduced regarding the effects of sound on the brain. Poor health is reported to be associated with lower parasympathetic tone in several medical conditions, including epilepsy [22]. Lotufo et al. report a sympathovagal imbalance in epilepsy, as shown by lower high frequency (HF), the standard deviation of the RR interval (SDNN), and the square root of the mean squared differences of successive RR intervals (RMSSD) values when compared to controls [23]. One study shows that a two-hour music intervention in cancer patients increases their relaxation scores and parasympathetic activities [24]. Another study shows that forty-five minutes of music therapy once a week in patients with cerebrovascular disease enhances parasympathetic activities and decreases congestive heart failure events by reducing plasma cytokine and catecholamine levels [25]. Our previous data also demonstrates that significant increases in HF, RMSSD, the standard deviation of differences between adjacent RR intervals (SDSD), and a decrease in mean beats per minute in heart rate variability analysis occurs while listening to Mozart music in children with epilepsy. At the same time, epileptiform discharges are significantly reduced during and right after listening to Mozart music. The results suggest that Mozart music stimuli induces parasympathetic activation [26]. It is possible that musical enhancement of parasympathetic tone may account for the beneficial effects on epilepsy.

Neurotransmitter pathways may also be involved in the effect of Mozart K.448 on epilepsy. Musical exposure is known to increase the expression of dopamine levels in the brain [2]. In recent years, the role of dopamine in the pathophysiology of epilepsy has been well documented. A Positron Emission Tomography study shows that impaired dopamine uptake in the midbrain is hypothesized to contribute to seizures in juvenile myoclonic epilepsy [27]. In a recent animal study, the authors report that pentylenetetrazole induced seizures decrease the dopamine levels in striatal and hippocampal areas, accompanying the induction and propagation of seizures [28]. It is possible that listening to music modifies the dopaminergic pathways contributing to the beneficial effects in epilepsy therapy.

There are limitations to this present study. First, the number of participants was somewhat limited and most of the patients were idiopathic in etiology. The statistical power is 0.74, under alpha error 0.05, based on 22 treatment and 24 control subjects with non-recurrence rates 0.63 and 0.23, respectively. Although the power is not sufficient, our findings may provide preliminary evidence that Mozart K.448 listening is beneficial for children with first unprovoked seizures. Second, the lack of control music made it impossible to say that the effects were specific to Mozart K.448 or to a placebo effect. Third, because we did not follow up the EEG in all patients in the control group, it could not be determined whether time duration itself could have caused the decreases in epileptiform discharges, although we had shown that there were significant decreases in epileptiform discharges after listening to Mozart K.448 in treatment group. Fourth, we used per protocol analysis with compliant patients instead of using intention-to-treat analysis, although the result of intention-to-treat is also significant (p = 0.029).

## Conclusions

In conclusion, listening to Mozart K.448 reduced the seizure recurrence rate and epileptiform discharges in children with first unprovoked seizures, especially of idiopathic etiology. Although there were limitations to the case numbers in this study, the results highlight that Mozart K448 listening is a promising alternative treatment in patients with first unprovoked seizures and abnormal EEGs. More investigations should be performed to substantiate the effects of music on first unprovoked seizures.

## Competing interests

The authors declare that they have no competing interests.

## Authors’ contributions

LCL carried out the study, participated in the evaluation of data, and helped draft the manuscript. MWL, RCW, and HKM conceived the study and participated in the evaluation of data. RCY participated in the design of the study, evaluation of data, and wrote the final version of the manuscript. All authors read and approved the final manuscript.

## Pre-publication history

The pre-publication history for this paper can be accessed here:

http://www.biomedcentral.com/1472-6882/14/17/prepub
